# Low follicular fluid tyrosine concentration in infertile women with ovarian hyperstimulation syndrome

**DOI:** 10.3892/br.2014.251

**Published:** 2014-03-12

**Authors:** NAMIKO AMANO, KOTARO KITAYA, SAGIRI TAGUCHI, MIYAKO FUNABIKI, YOSHIHIRO TADA, TERUMI HAYASHI, YOSHITAKA NAKAMURA

**Affiliations:** IVF Center, Oak Clinic, Osaka 557-0045, Japan

**Keywords:** dopamine agonist, follicular fluid, *in vitro* fertilization, ovarian hyperstimulation syndrome, tyrosine

## Abstract

The aim of this study was to compare the branched-chain amino acid (BCAA) and tyrosine concentration in the follicular fluid of infertile women with and without ovarian hyperstimulation syndrome (OHSS) in an *in vitro* fertilization program combined with controlled ovarian stimulation. Follicular fluid was aspirated during oocyte retrieval from 20 infertile patients who developed moderate-to-severe OHSS and 20 age- and body mass index-matched normoresponders. BCAA and tyrosine concentration were measured using enzymatic methods. The follicular fluid BCAA concentration was similar between the two groups (P=0.55), whereas tyrosine concentration was significantly lower in the OHSS compared to that in the normoresponder group (P=0.027) and the BCAA/tyrosine ratio was significantly higher in the OHSS compared to that in the normoresponder group (P=0.034). These results suggest an association between low follicular fluid tyrosine concentration and OHSS. Dopamine receptor agonists may be used as potential anti-OHSS medicines and tyrosine, as a dopamine precursor, may play a role against the development of OHSS.

## Introduction

Ovarian hyperstimulation syndrome (OHSS) is one of the serious complications that occur in ovulation induction cycles during infertility treatment or, occasionally, following conception. OHSS is characterized by cystic ovarian swelling and shifting of fluid from the circulation to the third space. Human chorionic gonadotropin plays a central role in the onset of OHSS. Human chorionic gonadotropin increases chemokine production in the granulosa and theca cells surrounding the ovulating oocytes. These chemokines bind to their specific receptors, expressed on local and systemic endothelial cells, which, in turn, transactivate vascular endothelial growth factor receptor-2 and its signaling pathway. This signaling stimulates polymerization of cytoskeletal actin and phosphorylation of adherens junction and tight junction molecules in endothelial cells. These molecular events induce conformational changes in the endothelial cells and vascular hyperpermeability. In its severe form, OHSS potentially leads to life-threatening conditions, such as massive ascites, pleural effusion, oliguria and thrombosis/embolism following hemoconcentration (1).

Infertile women with polycystic ovarian syndrome (PCOS) are at a high risk for developing OHSS in ovulation induction protocols (2). PCOS is also closely associated with obesity and insulin resistance. It was recently demonstrated that weight loss following gastric bypass surgery improved insulin resistance in obese patients with a decrease in circulating branched-chain amino acid (BCAA) levels (3). These findings suggest a possible association between BCAA and OHSS. The aim of this study was to investigate the follicular fluid BCAA level in infertile women who developed OHSS during controlled ovarian stimulation in an *in vitro* fertilization program.

## Materials and methods

### Subjects and methods

Between July, 2012 and September, 2012, follicular fluid was retrieved from infertile patients at the time of oocyte collection during *in vitro* fertilization/intracytoplasmic sperm injection cycles (4). All the patients had undergone controlled ovarian stimulation using gonadotropin-releasing hormone agonist (short protocol) or antagonist (flexible protocol) (5). The follicular fluid samples were immediately frozen at −80°C until measurement. Samples with macroscopic blood contamination were excluded from the investigation. A total of 20 patients who developed moderate-to-severe OHSS and 20 age- and body mass index-matched normoresponders were enrolled in the study. The follicular fluid concentration of BCAA (a total of leucine, isoleucine and valine concentration) and tyrosine (standardization control) were measured using an enzymatic assay (Diacolor-BTR kit; Toyobo, Osaka, Japan).

This study was approved by our Institutional Review Board (protocol no. 125081903) and all the subjects provided informed consent.

### Statistical analysis

The values were compared between the OHSS and the normoresponder group using the two-tailed Student’s t-test, non-parametric Mann-Whitney U test, or Pearson’s χ^2^ test.

## Results

### Demographics

There were no significant differences in demographics between the patients and the normoresponders, including infertility period, infertility category (85% primary infertility rate in the OHSS and 80% primary infertility rate in the normoresponder group) and the values of basal pituitary hormones and estradiol (Table I). However, the total human menopausal gonadotropin dose was significantly lower in the OHSS compared to that in the normoresponder group (P<0.0001). The number of the oocytes retrieved and fertilized and the number of the embryos/blastocysts frozen were significantly higher in the OHSS compared to that in the normoresponder group (P<0.0001). Fresh embryo/blastocyst transfer cycles were cancelled in all the patients with OHSS.

### Association of PCOS and OHSS

The incidence of PCOS was significantly higher in the OHSS (40%) compared to that in the normoresponder group (15%, P=0.0001). The number of oocytes retrieved, fertilization, blastocyst achievement and blastocyst freezing rates were also significantly higher in the OHSS compared to those in the normoresponder group (P<0.011), as were the cumulative clinical pregnancy and ongoing pregnancy rates within three transfer cycles (P<0.0004). There were no fatal complications in neither of the two groups.

### BCAA and tyrosine

**Figure 1:**
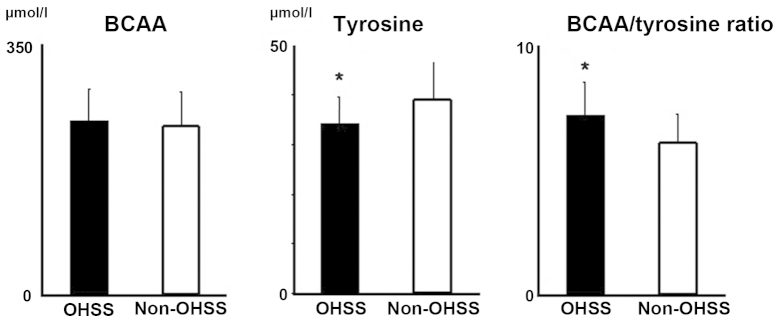
Comparison of follicular fluid branched-chain amino acid (BCAA) concentration, tyrosine concentration and BCAA/tyrosine ratio between patients with ovarian hyperstimulation syndrome (OHSS) and normoresponders in an *in vitro* fertilization program. Data are presented as means ± SD. ^*^P<0.05..

The follicular fluid BCAA concentration was similar between the two groups (P=0.55), whereas the tyrosine concentration was significantly lower in the OHSS compared to that in the normoresponder group (P=0.027, Fig. 1). Corresponding to these results, the BCAA/tyrosine ratio was significantly higher in the OHSS compared to that in the normoresponder group (P=0.034).

## Discussion

In this study, the follicular fluid BCAA concentration was found to be similar between infertile patients with and those without OHSS. In the Japanese population, the incidence of obesity in infertile women with PCOS is significantly lower compared to that reported in Caucasian populations (5). The incidence of PCOS in our cohort was significantly higher in the patients who developed moderate-to-severe OHSS compared to that in non-OHSS patients, whereas there were no obese women in the two groups. Thus, further investigations are required to compare follicular fluid BCAA concentration between obese and non-obese PCOS patients.

The follicular fluid tyrosine concentration, which was measured as a standard control protein, was found to be significantly lower in OHSS patients compared to that in normoresponders. In the human body, tyrosine is an amino acid which is synthesized from phenylalanine. The ovary is considered to be one of the active sites of tyrosine synthesis (6). Tyrosine is converted to L-3,4-dihydroxyphenylalanine by the action of hydroxylase and then to dopamine by aromatic L-amino acid decarboxylase.

Dopamine receptor agonists, such as docarpamine and cabergoline, are attracting increasing attention as promising OHSS-preventive agents (7). The binding of high-dose dopamine receptor agonists to dopamine receptor-2 was shown to interfere with vascular endothelial growth factor-mediated signaling by stimulating internalization of vascular endothelial growth factor receptor-2, decreasing its surface expression and rendering it unreachable to vascular endothelial growth factor (7). A decrease in the follicular fluid concentration of the dopamine precursor tyrosine in OHSS patients supports the hypothesis that tyrosine deficiency in growing follicles may lead to subsequent dopamine deficiency and dopamine receptor-mediated signaling attenuation, which may be involved in the etiology and pathogenesis of OHSS. The potential limitation of this retrospective study is the bias in participant selection. Although a larger sample size would be required to address this issue, the marked difference in this small cohort reflects the significance of the findings.

In conclusion, our results suggest an association between low follicular fluid tyrosine level and the onset of OHSS. As in the serum, tyrosine concentration in the follicular fluid is easily measurable in the clinical practice and may be a potential predictive marker of OHSS.

## Figures and Tables

**Table I:** Demographics and *in vitro* fertilization outcome in patients who developed ovarian hyperstimulation syndrome (OHSS) and in normoresponders.

Variables	OHSS^a^	Normoresponders^a^	P-value
Age (years)	34.0±3.3	33.4±3.9	0.60^b^
Body mass index (kg/m^2^)	20.7±2.1	20.1±1.8	0.64^c^
Infertility period (months)	31.0±26.3	24.9±11.7	0.26^b^
Basal luteinizing hormone level (IU/l)	4.9±2.5	4.4±3.0	0.58^c^
Basal follicle stimulating hormone level (IU/l)	5.2±1.5	5.4±2.1	0.75^c^
Basal estradiol level (pg/ml)	81.7±23.9	82.1±24.7	0.83^c^
Prolactin level (ng/ml)	14.1±5.7	16.5±9.7	0.33^c^
Total human menopausal gonadotropin dose (IU)	2,087.5±926.7	2,554.1±544.8	<0.0001^c^
Number of oocytes retrieved	17.6±6.0	7.3±2.4	<0.0001^b^
Number of oocytes fertilized	9.5±4.9	3.8±2.7	<0.0001^b^
Number of fresh embryos/blastocysts transferred	0	0.8±0.7	<0.0001^b^
Number of embryos/blastocysts frozen	6.1±4.2	1.9±2.4	<0.0001^b^

a Data are presented as means ± standard deviation.

b Mann-Whitney U test.

c Two-tailed Student’s t-test.
